# Room-temperature near-infrared up-conversion lasing in single-crystal Er-Y chloride silicate nanowires

**DOI:** 10.1038/srep34407

**Published:** 2016-10-05

**Authors:** Rui Ye, Chao Xu, Xingjun Wang, Jishi Cui, Zhiping Zhou

**Affiliations:** 1 State Key Laboratory of Advanced Optical Communication Systems and Networks, School of Electronics Engineering and Computer Science, Peking University, Beijing, 100871, China

## Abstract

Near-infrared up-conversion lasing in erbium(Er)-yttrium(Y) chloride silicate nanowires was demonstrated when pumped by 1476 nm laser at room temperature. The emission covers a very wide wavelength range (400–1000 nm). A clear threshold for 985 nm peak was observed at a launched average pump power of approximately 7 mW. Above threshold, the intensity increases linearly when turning up the pump power. The full width at half maximum at 985 nm decreases from 1.25 nm to 0.25 nm when reducing the measurement temperature from 30 K to 7 K, which is the narrowest linewidth of 980 nm micro-lasers to date. Our demonstration presents a possible novel method of utilizing up-conversion mechanism in Er-Y nanowire to achieve tunable near-infrared laser, which breaks new ground in the exploration of nanoscale optoelectronic devices operating at near-infrared wavelength.

Near-infrared lasers are significant for optical data communication, spectroscopy and medical diagnosis. Semiconductor nanowires, such as InGaAs nanowires, are superior in reducing the footprint of three-dimensional device and efficient as high-gain material in a wide range of wavelengths, and hence are being extensively studied in the field of visible, near-infrared and ultraviolet nanowire lasers^1^,^2^,^3^,^4^,^5^,^6^,^7^,^8^,^9^,^10^. However, these lasers usually need a shortwave high power laser as the pump source, which have a large volume and cannot be integrated to sub-micro and nanometer scale. The limitation is a big hindrance to the future implement of micro-laser chip. Er^3+^ ions, however, can absorb infrared photons in 1480 nm–1580 nm and emit visible band (400 nm–700 nm) or near infrared (980 nm) photon through up-conversion process at ^4^I_13/2_ level of Er^3+ ^11. In the recent years, the remarkable up-conversion (UC) phenomenon of Er^3+^ has been studied and applied in lasers^12^, solar cells^13^, analytical sensors, *in vivo* imaging^14^ and so on. However, the low UC luminescence efficiency is still one of the main limiting factors. To obtain a higher UC luminescence efficiency, the distance between the Er^3+^ ions needs to be very short. Usually, the doping concentration of Er^3+^ ions is very low because of the constraint of solid concentrations in conventional Er^3+^ doped silicon or silica materials (10^19^ cm^−3^). Therefore, erbium silicates, due to the high density of Er ions(~10^22^ cm^−3^), 100 times higher than that of other Er^3+^ doped materials, have attracted much interest recently^14^,^15^,^16^,^17^,^18^,^19^. The erbium silicates thin films have been demonstrated with a large propagation loss due to the lattice defects scattering, surface and side roughness induced during the micro-fabrication process^17^, which limits the further increase of UC efficiency. To reduce the propagation loss, Pan *et al*. fabricated the erbium silicates nanowires with a low propagation loss at 1530 nm due to high-quality single crystal nature^18^,^19^. However, UC luminescence properties of these erbium silicates nanowires are not yet studied up to now.

In this letter, we demonstrated the near-infrared up-conversion lasing in erbium(Er)-yttrium(Y) chloride silicate nanowires when pumped by 1476 nm laser at room temperature. The UC luminescence was observed with a wide wavelength range at 400–1000 nm. The 985 nm band emission due to the cooperative up-conversion of two 1480 nm photons, shows a much higher UC efficiency and has separated sharp emission lines with a full width at half maximum(FWHM) of only 0.25 nm under 77 K. A clear threshold for 985 nm peak was observed at a launched average pump power of approximately 7 mW at room temperature. Below threshold, the intensity increases exponentially with the pump power; Above threshold, it increases linearly when turning up the pump power. And saturation has not been observed with the maximum pump power of 115 mW. Yttrium ions(Y^3+^) were further added to the nanowires to adjust the distance between Er^3+^ ions and optimize the optical properties. The properties of Er/Y silicate nanowires indicate its lasing potential in achieving high-gain 980 nm nanowires optical waveguide amplifiers and lasers.

## Materials and Methods

The single-crystal Er-Y chloride silicate nanowires were grown by the chemical vapor deposition method. First of all, ErCl_3_ micro beads(Alfa Aesar, 99.99%) were mixed with YCl_3_ micro powder (Strem Chemicals, 99.9%) and silicon powder(Strem Chemicals, 99.999%) according to the proportion of atomic and placed in a ceramic boat at the heating zone of the furnace. Afterwards, a Si substrate (100) pre-coated with dispersed Au particles was positioned downwind with a distances of 10 cm away from the center of tube furnace to collect the deposited Er-Y chloride silicate nanowires. During the process, the quartz tube was heated to 1080 °C in 40 mins and maintained for 180 mins with a constant N_2_ gas flow of 70 sccm. The temperature of the Si substrate position was approximately 650 °C. In the end, the tube furnace was cooled to room temperature naturally.

**Figure 1:**
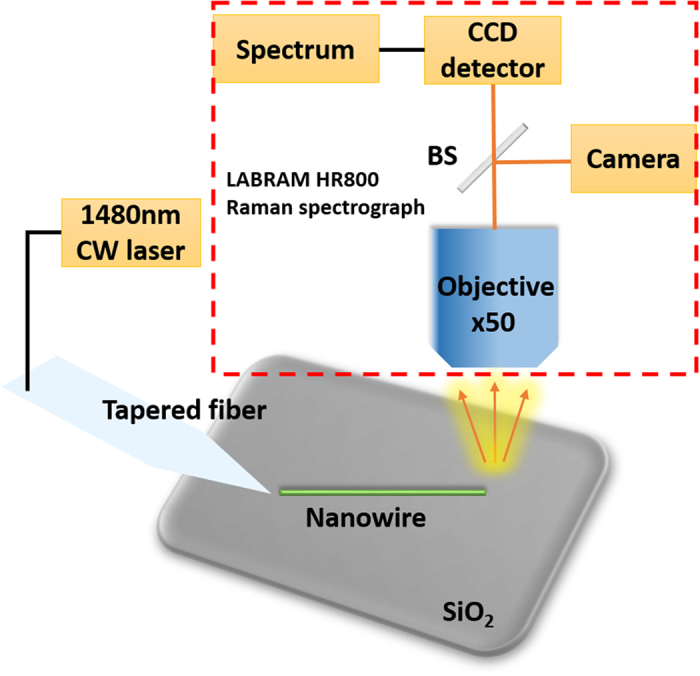
The schematic diagram of PL measurement system.

The scanning electron microscopy (SEM) images and *in situ* energy dispersive X-ray spectroscopy (EDS) analysis were performed using a Nova_NanoSEM430 equipped with an energy-dispersive X-ray detector. The high resolution transmission electron microscopy (HRTEM) images were collected by a Tecnai F20 Hi-Resolution transmission electron microscopy at 200 kV, equipped with a link EDS detector. X-ray diffraction (XRD) data were collected on the DMAX-2400 Materials Research X-ray Diffractometer. Photoluminescence (PL) was conducted using a home build Near Infrared PL system based on a LABRAM HR800 Raman spectrograph. A continuous wave laser at 1480 nm with a maximum power of 115 mW was focused to one port of the sample for optical excitation through a tapered fiber with a 3 μm tip. The PL signal was collected through the objective and finally detected by a liquid nitrogen cooled CCD detector, shown in Fig. 1. The low temperature PL measurement was carried out in a cryostat (Janis ST500).

## Results and Discussion

**Figure 2:**
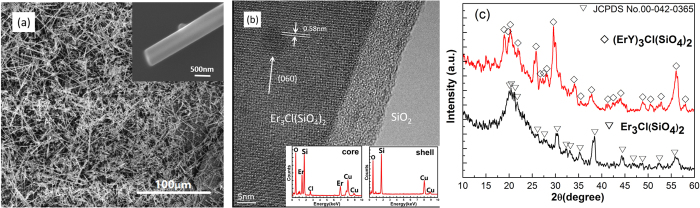
(**a**) SEM image of the as-grown ECS nanowires and amplified image of a representative nanowire (inset). (**b**) HRTEM image taken from the surface of the nanowire. Insets: EDS collected at the core and shell region. (**c**) XRD patterns of the ECS and EYCS nanowires.

Figure 2a shows the SEM image of the as-grown Er-Y chloride silicate nanowires. The wires with varied diameters (200 nm–900 nm) and tens of micrometers length in high yield were deposited on the Si substrate. The inset of Fig. 2a shows an amplified SEM image of a representative nanowire with a diameter of about 650 nm. The single nanowire has a uniform diameter and a highly smooth surface, which can help to decrease the scattering light into the air at the surface. Figure 2b shows the HRTEM image taken at the surface of the sample. A large dark-bright contrast variation between the middle and outer sections indicates that the wire consists of the different masses materials. The TEM-EDS spectra collected from the darker and lighter interior regions are shown in the inset of Fig. 2b, respectively. The spectra of the core section shows peaks of elements Er, O, Cl, Si and Cu(from the copper grid), while that of the shell section only shows peaks of elements O, Si and Cu. It indicates that the investigated nanowire body consists of a highly crystalline core covered by a 10 nm-thick amorphous silicon oxide shell. The core has a lattice spacing of 0.58 nm, consistent with the {060} inter-planar distance of orthorhombic structure of Er_3_Cl(SiO_4_)_2_ (ECS). Figure 2c shows the XRD patterns of the Er chloride silicate nanowires and Er-Y chloride silicate nanowires, respectively. The signal collected from the nanowires is tiny and in the same order of magnitude to the noise, so the curves encountered a manual smooth processing to help detect characteristic peaks. The peaks marked with triangle and diamond markers are well indexed with an orthorhombic crystal ECS (JCPDS card: No. 00-042-0365). The Er_3−x_Y_x_Cl(SiO_4_)_2_ (EYCS) samples synthesized from the different amount of ErCl_3_ and YCl_3_ presented similar XRD features with that of ECS. The small difference in size between Er^3+^ and Y^3+^ions could result in a small change of the lattice constant.

**Figure 3:**
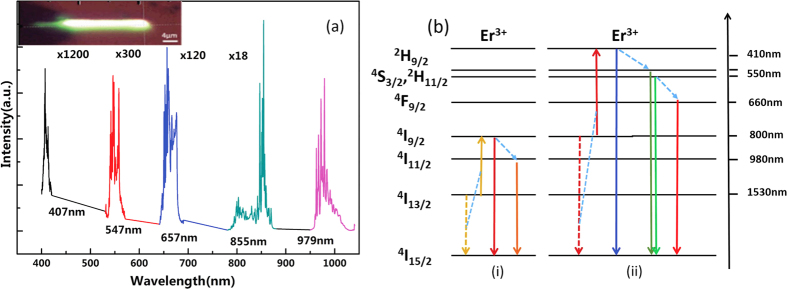
(**a**) Up-conversion light emission spectra at visible and near infrared wavelength range. Inset: optical image of the pumped nanowire; (**b**) Erbium ion energy level involved in up-conversion emission. (i) The first-order CUC. (ii) The second-order CUC.

Figure 3a shows the measured nanowire’s UC spectra at 400–1050 nm when pumped by 1480 nm laser at a power of 115 mW and its optical image (inset). The center wavelengths of the peaks are shown below the curves. The linewidths for 407 nm, 547 nm, 657 nm, 855 nm and 979 nm are 0.5 nm, 1.25 nm, 1.25 nm, 1 nm and 1.25 nm, respectively. The measured nanowire in this experiment has a diameter of 900 nm and a length of 20 μm. The distance between fiber tip and nanowire port was adjusted to get the best coupling efficiency. Bright green light spreading over the sample was observed from the image. However, as shown in the spectra of Fig. 3a, the dominant UC emission arises at 980 nm, which has intensity more 300 times than that of green UC. The 980 nm UC emission arises from the two photon cooperation up-conversion (CUC), while the green UC emission arises from the cooperation of three photons and more, which is more complicated and hence much harder to happen. Figure 3b shows the Er^3+^ energy levels involved in UC emission. At 1480 nm, pumping directly excites the upper sublevels of the ^4^I_13/2_ metastable manifold (the excited state is broadened into closely spaced sublevels due to Stark splitting), with ground to excited state transition (^4^I_15/2_ → ^4^I_13/2_), corresponding to both a 1520 nm–1570 nm signal band and 1460 nm–1500 nm pump band. Figure 3b(i) depicts the first-order CUC process, which explains 850 nm and 980 nm light emission. Though both 880 nm and 980 nm band emission come from the first-order CUC, but the lifetime of photons at ^4^I_9/2_ is much shorter than that of ^4^I_11/2_, so the photons get through a rapid relaxation and accumulate at ^4^I_11/2_ in abundance. So the intensity of 980 nm emission is more 18 times than that of 850 nm emission. Then photons at ^4^I_9/2_ state encounter another CUC process and emit 410 nm light (blue), 550 nm light (green), 660 nm light (red), shown in Fig. 3b(ii). Increasing the pump power gradually, the intensity of 985 nm and 860 nm light grows rapidly. After the pump power has been increased to about 25 mW, the light at the short wavelength band arises while the increasing rate of 860 nm and 985 nm remains the same. This further demonstrates that the emission at visible band comes from the second-order CUC process of photons at ^4^I_9/2_ state.

**Figure 4:**
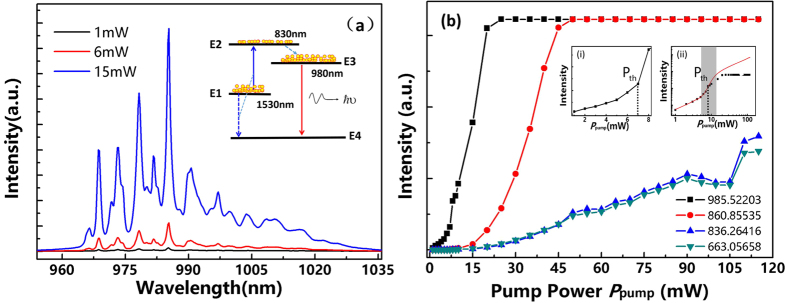
(**a**) Spectra at 950–1035 nm band under different pump power and the corresponding four energy state model (inset). (**b**) The dependence of the integrated intensity on the pump power; (i) Magnified view around the threshold point. (ii) Nonlinear response of laser output power with increasing pump power.

Figure 4a shows the spectra of the nanowire around 980 nm pumped by 1480 nm laser on 1 mW, 6 mW, 15 mW, respectively. As previously discussed, the nanowires are highly ordered and crystalline, as a result, obvious multi-peaks with high signal to noise ratio in the spectra are observed even at very low pump power (1 mW). The FWHM of four main peaks is almost the same (~1.25 nm) and no obvious change in the value was observed when increasing the pump power. The similar single or multiple sharp peaks at the visible wavelengths were observed in Yang’s work, which explain them as different lasing modes^20^,^21^. The inset shows the corresponding four energy state model of active Er^3+^ ions, in which the Er^3+^ ions firstly absorb 1480 nm photons and accumulate at ^4^I_9/2_ state in abundance, which serves as the E1, then transmit to higher excited state E2 through the following UC process, after going through a rapid relaxation to lower excited level (E3), finally execute a downward transition to E4 and give out the lasing. The process is similar to the traditional four energy state model for semiconductor lasers. The lasers using four energy state model are known for low threshold due to no electron accumulation in E4 and hence easy to realize population inversion condition. Similar to the traditional one, no much electrons accumulate at E4 and hence a low threshold is expected.

The dependence of the integrated intensity on the pump power at different wavelengths is presented in Fig. 4b. All these curves share the same tendency. A clear threshold for 985 nm peak was observed at a launched average pump power of approximately 7 mW, shown in left inset. Below threshold, only amplified spontaneous emission is to be seen, which increases exponentially with the pump power. Above threshold, the output power increases linearly with the pump power. In our experiment, the saturation of the lasing power has not yet been observed. For very high pump powers, the laser power meets the maximum detection power limitation. However, in view of the curves for 860 nm, 836 nm, and 663 nm, potentials for further large augment are indicated and saturation has not been observed at the maximum measuring pump power, which indicates that no obvious thermal damage was induced in the nanowire so far. The right inset presents nonlinear response of laser output power with increasing pump power plotted on a double-log scale, showing threshold region as a ‘kink’ between the two linear regimes of spontaneous emission and lasing. The grey area is the region of amplified spontaneous emission. The good lasing properties can be attributed to the existence of nanowire cavity. The light is first pumped at the port without gold, then propagates and gets reflected in the nanowire, after several circles of propagation and mode-selection, finally gets out from the same port. The refractive index difference (between gain material Er-Y Silicate (n = 1.8) and air (n = 1)) and the wire-like geometry enable strong two-dimensional confinement of photonic modes guided along the nanowire axis, and the end facets (one port with gold) provide superb optical feedback for these guided modes.

**Figure 5:**
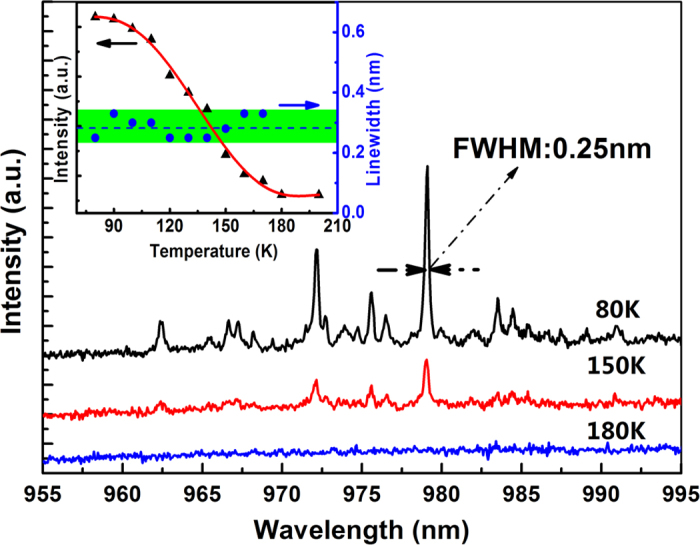
Spectra of the nanowire around 979 nm in different measurement temperature and the dependence of emission intensity and linewidth of the 979.1 nm peak to the temperature (inset).

However, due to the thermal effect in the nanowire, the four main peaks in the 980 nm spectra are not completely separated from each other and the spectrum around the peaks increases at the same rate as the pump power. To further analyze the UC emission properties and exclude the influence of the thermal effect, we retest the spectrum property in the same nanowire under 77 K (liquid nitrogen). Figure 5 presents the spectra of 980 nm band at different measurement temperature of 80 K, 140 K, 180 K pumped by space coupling at a maximum pump power of 225 μW. It is noted that the FWHM of the peak around 979.1 nm has an ultra-narrow value of 0.25 nm. The inset of Fig. 5 shows the intensity and linewidth variation with the increase of the temperature. The intensity reaches its highest value at 77 K and then keeps decreasing when further increasing the temperature to 180 K. The peaks are finally overwhelmed by the background due to the limitation of the maximum pump power. The value of the linewidth keeps stable between 0.25–0.33 nm, indicating the crystal lattice constant did not change. Combined with the results measured at room temperature, a narrow-linewidth 980 nm lasing with 1000-times-higher intensity is expected when the nanowire can be pumped by 110 mW or more at 77 K.

The proportion of the raw material (ErCl_3_ and YCl_3_ micro-powder) was changed to get the different Er_x_Y_3−x_Cl(SiO_4_)_2_ nanowires with x = 3, 1.5, 0.6, 0.4, in order to further analyze the role of Y^3+^ in reducing the concentration quenching. A blue shift of the emission wavelength was observed when more Y^3+^ ions were doped into the Er silicate. The wavelength of the strongest peak varies more than 10 nm (from 984.4 nm to 973.0 nm), which can be mainly attributed to two factors. Firstly, a slight increase in lattice constant occurred when Y^3+^ substitute a portion of Er^3+^ in the lattice (the lattice constant of Y is 0.90 Å and that of Er is 0.88 Å). Secondly, the distance between Er^3+^ ions is enlarged by adding Y in the silicate, so the band bending due to the interaction of the different Er^3+^ ions is reduced. It is also possible that the slight difference in geometric dimensioning of the different nanowires measured would have an influence on the peak wavelength, which may be demonstrated in future works. A slight variation was observed in the intensity ratio between different peaks when increasing the pump intensity, which is due to the band gap renormalization. The variation of the peak wavelength induced by adding of Y^3+^ in the nanowires presents an available method towards the implement of tunable 980 nm LED or laser in the future. Moreover, when pumped at the same power, the sample(x = 1.5) showed the highest emission intensity at 980 nm, which indicates an optimal UC efficiency.

The low refractive index difference between EYCS (n = 1.68^22^,^23^,^24^,) and the SiO_2_ substrate (n = 1.4) or air (n = 1.0) induces a very low limiting factor and there exists large leakage loss to the substrate or air. To enhance the limiting factor, several ways can be adopted. For example, by embedding the EYCS nanowires into high-index silicon, the radiative efficiency of Er^3+^ in the double slot waveguides will be significantly improved^25^,^26^,^27^. Building an external cavity can provide light feedback, which will also help to enhance the proportion of the remaining light in the nanowires^27^,^28^,^29^,^30^,^31^,^32^. What’s more, in view of the high-intensity radiation over a wide wavelength range, there is a tremendous opportunity to achieve an effective and cheap monolithic white laser by adopting three parallel-placed Er-Y silicate nanowires if an additional grating or photonic crystal is etched and integrated into the surface of the nanowires to help separate light from blue, yellow and red band, respectively^33^,^34^,^35^. All these methods route towards efficient EYCS nanowires based micro-laser in the future work.

## Conclusion

In conclusion, the 980 nm lasing properties of single-crystal Er-Y chloride silicate nanowires with ultra-narrow linewidth was demonstrated when pumped by 1476 nm laser. Well separated sharp emission lines within the visible and infrared band have a linewidth of only 0.25 nm at 77 K. A clear threshold for 985 nm peak was observed at a launched average pump power of approximately 7 mW. The superb linear relationship above threshold between emission intensity and pump power were presented. A two-order CUC process and a four energy state model was proposed and demonstrated to explain 980 nm up-conversion lasing mechanism. The Er/Y ratio in the nanowires was further varied to analyze the 980 nm up-conversion emission mechanism and a blue shift of the strongest peak in the band of the nanowires with higher ratio of Er/Y was observed. These 980 nm lasing properties of Er-Y chloride silicate nanowires presented above pave a new way of utilizing up-conversion mechanism in Er-Y nanowire to achieve tunable near-infrared laser and indicate its potential in future application in nanoscale optoelectronic devices operating at near-infrared wavelength.

## Additional Information

**How to cite this article**: Ye, R. *et al*. Room-temperature near-infrared up-conversion lasing in single-crystal Er-Y chloride silicate nanowires. *Sci. Rep.* **6**, 34407; doi: 10.1038/srep34407 (2016).
